# Time-Lapse GPR Measurements to Monitor Resin Injection in Fractures of Marble Blocks

**DOI:** 10.3390/s23208490

**Published:** 2023-10-16

**Authors:** Luigi Zanzi, Marjan Izadi-Yazdanabadi, Saeed Karimi-Nasab, Diego Arosio, Azadeh Hojat

**Affiliations:** 1 Dipartimento di Ingegneria Civile e Ambientale, Politecnico di Milano, 20133 Milan, Italy; luigi.zanzi@polimi.it; 2 Department of Mining Engineering, Shahid Bahonar University of Kerman, Kerman 76188, Iran; marjaniz326@gmail.com (M.I.-Y.); kariminasab@uk.ac.ir (S.K.-N.); 3 Department of Hydrology and Hydrodynamics, Institute of Geophysics Polish Academy of Sciences, Księcia Janusza 64, 01-452 Warszawa, Poland; 4 Dipartimento di Scienze Chimiche e Geologiche, Università degli Studi di Modena e Reggio Emilia, 41125 Modena, Italy; diego.arosio@unimore.it

**Keywords:** nondestructive testing, ground-penetrating radar, high-frequency antenna, marble, fracture characterization, epoxy resin injections, quality control

## Abstract

The objective of this study is to test the feasibility of time-lapse GPR measurements for the quality control of repairing operations (i.e., injections) on marble blocks. For the experimental activities, we used one of the preferred repairing fillers (epoxy resin) and some blocks from one of the world’s most famous marble production area (Carrara quarries in Italy). The selected blocks were paired in a laboratory by overlapping one over the other after inserting very thin spacers in order to simulate air-filled fractures. Fractures were investigated with a 3 GHz ground-penetrating radar (GPR) before and after the resin injections to measure the amplitude reduction expected when the resin substitutes the air. The results were compared with theoretical predictions based on the reflection coefficient predicted according to the thin bed theory. A field test was also performed on a naturally fractured marble block selected along the Carrara shore. Both laboratory and field tests validate the GPR as an effective tool for the quality control of resin injections, provided that measurements include proper calibration tests to control the amplitude instabilities and drift effects of the GPR equipment. The method is accurate enough to distinguish the unfilled fractures from the partially filled fractures and from the totally filled fractures. An automatic algorithm was developed and successfully tested for the rapid quantitative analysis of the time-lapse GPR profiles collected before and after the injections. The whole procedure is mature enough to be proposed to the marble industry to improve the effectiveness of repair interventions and to reduce the waste of natural stone reserves.

## 1. Introduction

In recent years, the quarrying industry has experienced increasing demands for non-destructive sensing techniques to optimize stone production, from on-site extraction operation, to quality control of the extracted blocks [1,2,3,4,5,6,7]. Fractures present in building stones (either in situ large fractures or small defects of the produced stones) have always concerned the quarry people as well as engineers engaged in the restoration and conservation of stone monuments. Fractures might extend in all three dimensions, and they play a key role in choosing the size and the shape of the extracted blocks as well as defining the maintenance strategies. Therefore, most introduced sensing technologies mainly focus on mapping the fractures [5,6,7,8,9,10,11,12]. Reliable characterization of rock fractures can considerably optimize the production of building stones. Moreover, it is helpful to develop techniques to map fractures in order to protect the stones currently in use in historical buildings and monuments and to be able to monitor the repair operation and check the condition of the stone after treatment.

The ground-penetrating radar (GPR) method is a totally non-destructive sensing technique capable of providing high-resolution images for a variety of engineering applications [13,14,15,16,17]. The GPR method has proved its ability as a valuable geophysical technique to enter the quarrying and construction industries to map the fractures at different stages: during exploration and extraction in quarries to reduce the production costs and wastes [6,7,10,11,12,18,19,20], and for post-production demands to repair and restore the stones [21]. From various research studies that use the GPR method to study rock fractures, we can mention the theoretical models and laboratory experiments used to characterize rock discontinuities [18,19,20,21,22,23,24,25,26], in situ GPR surveys to detect fractures at different zones of quarries or in historical buildings [7,10,12,15,22,27,28,29,30], and 3D GPR acquisitions for 3D reconstruction of the fractures and other features [11,12,31,32,33,34]. Most of these studies are focused on the application of the GPR method to detect rock/stone fractures to optimize the quarrying activities and to preserve the constructions. In recent years, an increasing interest emerged to explore the efficiency of the GPR method to diagnose fractures and determine their aperture and filling material. This is still an interesting research issue with ongoing studies [10,18,19,20,21].

Fractures observed at GPR frequencies can be generally considered as thin layers because the thickness of the fracture is normally much smaller than a quarter of the wavelength, which is the standard resolution limit, i.e., the minimum thickness that GPR can resolve. When the fracture thickness is larger than the resolution limit, the Fresnel reflection equation quantifies the amplitude and phase distribution of the waves separately received from both sides of the layer interfaces. On the contrary, when the fracture is a thin layer, the reflections from the two interfaces overlap in such a way that a single composite wavelet is received. The response of the thin layer is the sum of the primary reflections and multiple reflections that occur within the layer. Their interferences may be constructive or destructive and generate complex patterns. For layers much thinner than the wavelength, the value of the resulting reflection coefficient changes linearly with the thin bed thickness. When the layer becomes thicker, the magnitude of the reflection coefficient progressively grows with a decreasing gradient until it arrives at its maximum value in correspondence of a fracture aperture equal to a quarter of the wavelength. As a result, the reflection coefficient variations can be used for characterizing the thin bed properties [18,35,36,37,38,39], e.g., to study thickness variations or to distinguish different filling materials [18,21].

In the past, the most common solution to the problem of fractures was to replace the damaged stone with a new one. This method could negatively affect the beauty or the strength of a structure, especially for historic buildings or sculptures [8,40]. New strategies try to repair the damaged stones in a fast and high-quality way in order to overcome the disadvantages of old solutions. The repairing methods apply different techniques such as cement filling, injection of epoxy resin, polyesters resin, ultra-viola resin, and mastics [41,42] for the restoration and strengthening of building stones, especially decorative stones or the facades of historic buildings. The use of fillers for improving the consistency of building stones and repairing fractures of decorative stones has been discussed in various studies [43,44,45,46]. Proper injection of the filler into fractures will guarantee long-term protection of the stones. The choice of the method and the material with which to fill the fractures depends on several factors, such as the ratio of the cracks to the pores, the value and price, the color, and the application area of the stone [42,47]. The properties of epoxy resin have made it a widely-used solution for conserving and consolidating stones such as granites and marbles [48,49,50]. The epoxy resin is composed of an epoxide component and a hardener or curing agent and has been applied in the repair and preservation of a number of famous stone monuments in the world, especially in Italy [45]. Epoxy resins are thermosetting adhesive materials that are known for their strength and creep resistance and are widely used in the conservation and consolidation of stone, glass, and wood [51]. Epoxy resin typically has a good resistance to moisture and chemical agents especially alkali materials. Additionally, its excellent resistance to mechanical stress and its relatively high viscosity are two of the most significant characteristics of such resins [46,50]. Although epoxy resins are viscous, they can efficiently penetrate into porous and fractured zones. All the aforementioned benefits explain the reason why epoxy resin is an increasingly preferred choice to repair the fractures of stones and masonry structures [46]. Therefore, we used epoxy resin as the filler material to fill the fractures in this study. The GPR measurements were performed on air-filled artificial and natural fractures, and the measurements were repeated after filling the fractures with epoxy resin to assess the effectiveness of time-lapse GPR tests for quality control of the resin injections. Quality control is fundamental to ensure the complete penetration of the resin to fully fill the fractures, even at its most remote angles.

## 2. Materials and Methods

The objective of the experimental activities is to validate an effective procedure to collect and compare GPR measurements before and after fracture repair with the injection of epoxy resin. The final goal is to assess that the resin has fully sealed the fracture. According to the thin bed theory, the amplitude of the reflection of an electromagnetic wave from an air-filled fracture is expected to be higher than the amplitude of the same reflection after resin injection. However, time-lapse measurements should be properly executed and analyzed to appreciate the amplitude decrease resulting from injection. The amplitude is expected to be reduced by about 33% or even more [21]; the precise value depends on the permittivity values of the resin and the marble. 

### 2.1. Marble Specimens

**Figure 1:**
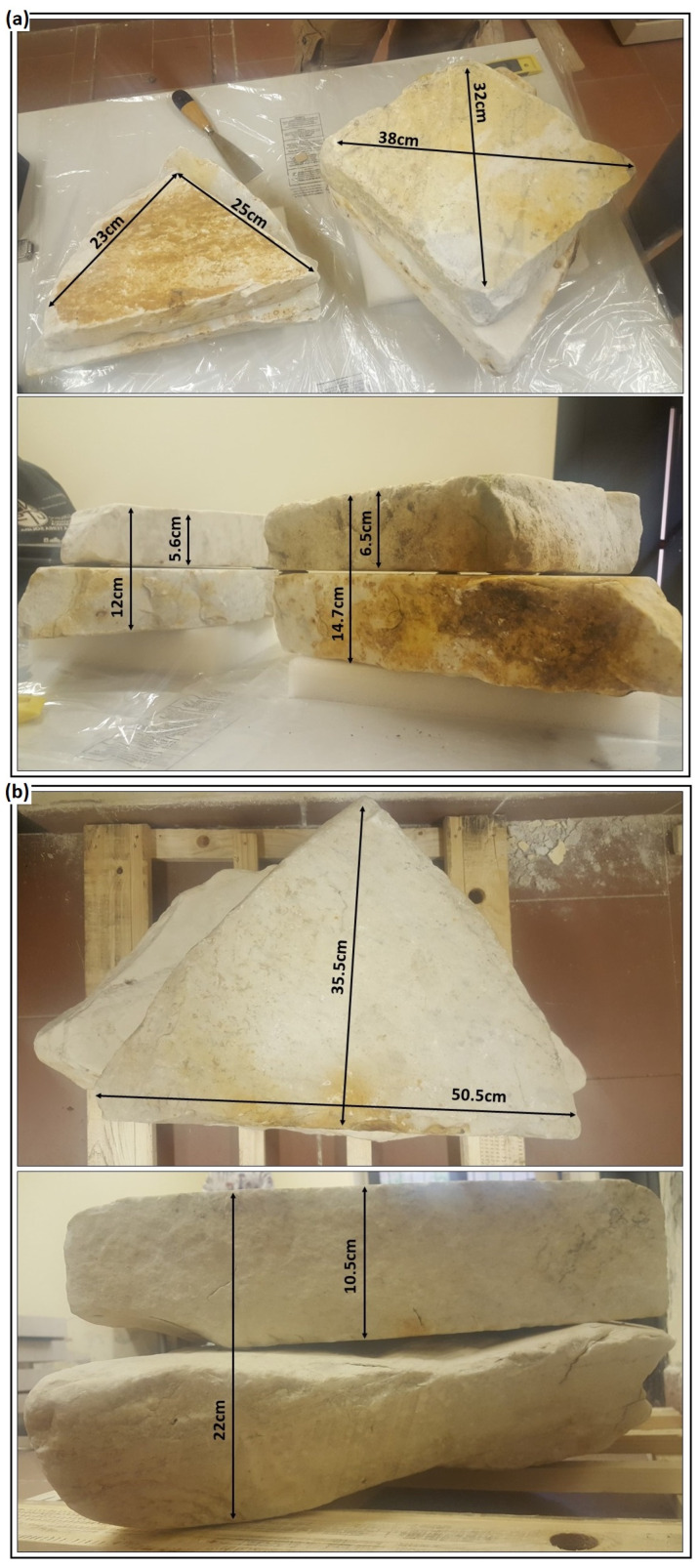
Top and side views of three fractured samples created for laboratory tests using six Carrara marble blocks. (**a**) Two small blocks (**left**) with the fracture modelled between them at the depth of about 5.6 cm with the average fracture opening of 3 mm. Two medium blocks (**right**) with the fracture modelled between them at the depth of about 6.5 cm with the average fracture opening of 3 mm. (**b**) Two large blocks with the fracture modelled between them at the depth of about 10.5 cm with the average fracture opening of 8 mm.

For laboratory tests, we used six small marble blocks selected from quarry wastes in Marina di Carrara, Italy. Using these six blocks, we prepared three specimens, shown in Figure 1, with slightly increasing dimensions. We created artificial and irregular fractures by placing the blocks on each other in pairs. To create artificial fractures, small PVC spacers with the height of 2 mm for small blocks (Figure 1a, left), 3.5 mm for medium blocks (Figure 1a, right), and 5 mm for large blocks (Figure 1b) were placed between each pair of blocks. The PVC spacers were positioned as close as possible to the edges in order to prevent their influence on the integrity of the resin after being dried. The inner surfaces of the blocks were not smooth or polished. As a result, the distance between the two blocks, i.e., the thickness of the simulated fracture, was not constant, especially for the large blocks (side view in Figure 1b) where the fracture thickness approximately varied between 1 mm and 15 mm. Based on the surface area of the blocks (Figure 1) and the volume of the resin required for the full injection, we calculated an average fracture opening of 3 mm for small blocks, 3 mm for medium blocks, and 8 mm for large blocks. Considering that the relative permittivity of the epoxy resin was expected to vary in the range 3.5–4.7, a quarter of the wavelength for the 3 GHz antenna used in this study was never less than 15 mm and the simulated fractures can be thus considered as thin layers. All specimens were surveyed with the GPR before and after resin injections in the laboratory.

In laboratory tests, we also used four uniform marble blocks with the dimensions 40 cm × 40 cm × 10 cm dedicated to this study from a Botticino classic marble quarry in Brescia province, Italy [52]. The main purpose of using regular Botticino marble specimens was to control the amplitude drift of the GPR antenna during measurements. 

**Figure 2:**
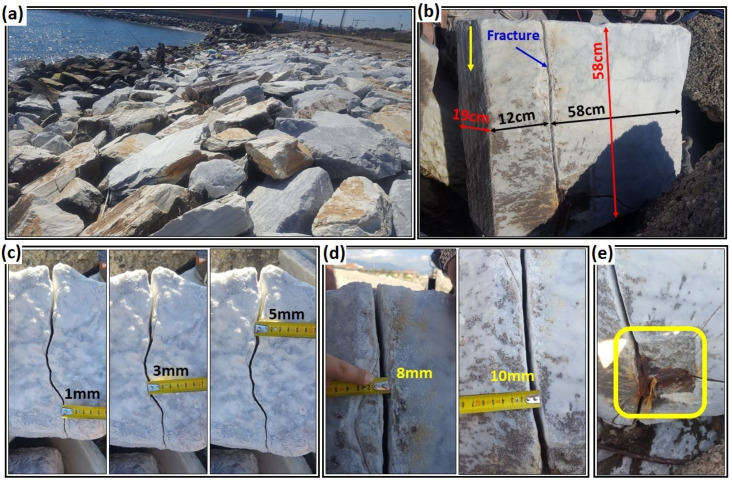
Carrara marble block for field test. (**a**) A pile of abandoned blocks on the Marina di Carrara coast. (**b**) The selected block with a vertical, visible natural fracture. The vertical yellow arrow shows the side selected for the GPR measurements. (**c**) Top view of fracture thickness. (**d**) Front view of fracture thickness. (**e**) Detail of a metal target embedded near the fracture in the lower part of the block.

We performed in situ measurements on a fractured marble block in Marina di Carrara where hundreds of irregular marble blocks were deployed on the shore to form a protecting barrier (Figure 2a). The selected block had the advantages of having a clearly visible air-filled fracture and an accessible side parallel to the fracture suitable for radar measurements (Figure 2b–d). Unfortunately, there was a small metal target embedded in the lower part of the block (Figure 2e), maybe related to a previous use of the block or used for block transportation. We performed time-lapse GPR measurements at the same locations before and after resin injections to repair the natural fracture of the selected block. As can be seen, the metal element shown on Figure 2e partially disturbed the radar measurements performed near the base of the block. 

**Figure 3:**
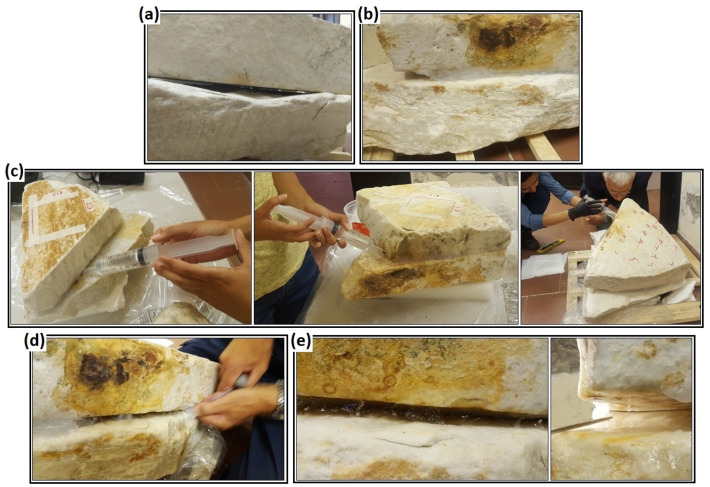
The process of simulating artificial fractures and resin injection in marble specimens. (**a**) Simulating a fracture by overlapping two blocks. (**b**) Sealing the fracture perimeter with silicone. (**c**) Resin injections inside the small blocks (**left**), medium blocks (**middle**) and large blocks (**right**). (**d**) Removing the dried silicone from around the blocks. (**e**) Checking that resin arrived at all the peripheral parts of the fracture.

To prevent the dispersion of the resin out of the fractures, both the artificial fractures between the overlapped blocks in the laboratory and the natural fracture in Marina di Carrara were properly sealed with silicone along their external perimeter with the exception of a small injection point (Figure 3).

### 2.2. GPR Measurements

The GPR equipment consists of an IDS system [53] with a 3 GHz antenna. All laboratory measurements were repeated with two different control units to compare the effect of their electronic circuits on the antenna amplitude drift.

**Figure 4:**
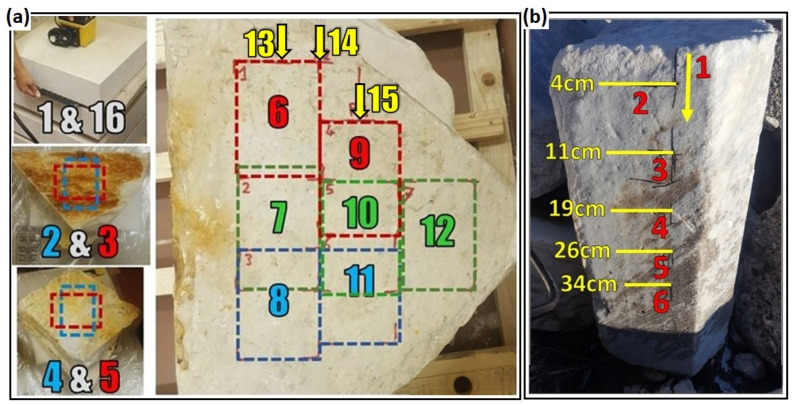
(**a**) The positions of the GPR antenna for different measurements performed in the laboratory. Records 1 and 16 were surveyed on marble blocks of Botticino for amplitude calibration at the beginning and end of each experiment. Records 2 and 3 were surveyed from the central position of the small blocks of Carrara (see Figure 1a, left). The antenna was rotated 90 degrees from record 2 to record 3. Similarly, records 4 and 5 were surveyed from the central position of the medium blocks of Carrara (see Figure 1a, right). Records 6 to 12 were surveyed on the large blocks of Carrara (see Figure 1b) by deploying the antenna at the fixed positions from 6 to 12. Profiles 13, 14 and 15 were recorded as continuous profiles parallel to the longer side of the blocks by moving the antenna from the positions 13, 14, and 15. (**b**) Measurements on the Carrara marble block with natural fracture used for field test (see Figure 2b). Profile 1 was recorded as a continuous profile from top to down. Records 2 to 6 were surveyed by placing the antenna at the positions 2 to 6, and yellow numbers are the distances of the antenna’s positions from the top of the block.

GPR measurements were performed on the laboratory specimens for eleven days. In addition, measurements on the Botticino blocks were performed every day for amplitude calibration. Each set of measurements consisted of 16 records. Two records (n. 1 and n. 16) were surveyed on Botticino marble blocks at the beginning and the end of the measurements of each day to control the amplitude drift of the radar system. The reference signal used to control the antenna amplitude drift was the reflection from a metal shield inserted between the first and the second blocks measured by the antenna from the central position of the first block. The remaining fourteen records were surveyed at the positions shown in Figure 4a. All GPR measurements were performed as time-triggered surveys without moving the antenna from the fixed position, except for records 13, 14, and 15 that were surveyed as wheel-driven profiles by moving the antenna on the large blocks along short trajectories. Profiles 13 and 14 were about 18 cm and 22 cm long, respectively, while profile 15 was about 12–14 cm long. 

The first three days were used to repeat the whole set of measurements on the air-filled fractures in order to check the repeatability and stability of the results. Resin injection started after the measurements of the third day and GPR measurements were repeated with the presence of resin inside the fractures for the following eight days. More information about the volume of the injected resin on each day is listed in Table 1. Injections were always performed after the GPR measurements of that day. The fractures of the small and medium blocks were fully saturated with resin with the first injection performed on the third day. The fracture created between the large blocks was progressively saturated with four injections. The large blocks were slightly dipping in such a way that the resin, injected from the block corner closer to position 6 (Figure 4a), was gently flowing by gravity towards the block side opposite to the injection point.

The GPR field activities on the fractured marble block selected along the Carrara coast started with a preliminary velocity test performed by collecting five time-triggered records around the block in the presence of a metal shield at the opposite side of the antenna. Velocities obtained as twice the block thickness divided by the shield reflecting time were averaged among the five results to minimize the measurement errors. Six other measurements were carried out to record the response of the air-filled and resin-filled fracture to the radar signal. The first profile was a continuous vertical profile about 40 cm long moving from top to bottom, and the other five profiles were time-triggered records. Figure 4b illustrates the approximate position of the antenna on the block for GPR tests. The perimeter of the fracture was then sealed with silicone, except for a small part to be used for resin injection from the top of the block. After the silicone had cured, we injected some epoxy resin into the fracture. The prepared amount was not enough to entirely fill the fracture. So, we decided to perform a first set of measurements after this partial injection was dried. Then we completed the injection by filling the fracture until no other resin could enter, and when the new injection was also dried, we repeated the set of measurements. Any set of measurements consisting of 6 records (one wheel-driven vertical profile and five fixed-position time-triggered profiles) was repeated at the positions illustrated in Figure 4b. Additionally, any set of measurements was accompanied by three velocity tests repeated in the three best positions already tested during the first measurement day; the objective of this being to collect the data suitable for antenna power calibration.

On the whole, for all seven Carrara marble blocks used in this study (either in lab or in the field), the wave velocity obtained from the velocity tests was 10.8 cm/ns, corresponding to a relative permittivity of 7.7.

### 2.3. Data Processing

The data acquisition and processing techniques of GPR measurements depend on the specific objective of each survey and the desired result. In this study, measured datasets were processed in ReflexW software, version 9.5.6 [54]. The purpose of data processing for the fixed-point measurements was to obtain a single stack trace from each record to determine the amplitude reflection of the thin bed.

After time calibration, a dewow filter was applied to remove the DC and the very low-frequency components affecting the records. Then, we applied a Butterworth bandpass filter to suppress the noise outside the bandwidth of the antenna. Finally, we compressed each time-triggered B-scan into a single A-scan by stacking all the traces belonging to the B-scan.

As mentioned, additional calibration tests were performed on the Botticino marble blocks at the beginning and at the end of each day of the laboratory measurements. The objective of the data processing of these tests was to extract the amplitude of the reflection produced by the metal shield at the base of the upper Botticino block (Figure 4a) in order to control any possible drift of the transmitted energy either during the day or from different measurement days. Thus, we processed these data with the same processing sequence described above; then, by comparing the reflected amplitude at the beginning and at the end of the day with an invariant reference amplitude, we extracted the calibration factor needed to remove the drift effect from the measurements on the Carrara blocks. The calibration factor was interpolated between the initial and the final calibration test of the day to redistribute the drift effect throughout the duration of the experiment. As seen on Figure 4a, each single day experiment consisted of 16 records and thus, the time span was split into 15 intervals, assuming that the acquisition of any GPR record was approximately taking the same time. As a result, the calculation of the calibration factor was performed according to the following equations:

(1) $${CF}_{1}=\frac{K}{{Amp}_{1}}$$

(2) $${CF}_{16}=\frac{K}{{Amp}_{16}}$$

(3) $${CF}_{i}=\left({CF}_{1}\right)-\left({CF}_{1}-{CF}_{16}\right)\times \frac{\left(i-1\right)}{15}$$

(4) $${CAmp}_{i}={CF}_{i}\times {Amp}_{i}$$

where K is an arbitrary value, ${CF}_{1}$ is the calibration factor of the first record, ${Amp}_{1}$ is the amplitude of the metal shield reflection of the first record, ${CF}_{16}$ is the calibration factor of the last record, ${Amp}_{16}$ is the amplitude of the metal shield reflection of the last record, i is the record number for Carrara blocks (ranging from 2 to 15), ${CF}_{i}$ is the calibration factor of the *i*th record, ${Amp}_{i}$ is the amplitude of the thin bed’s reflection of the *i*th record, and ${CAmp}_{i}$ is the same amplitude after calibration. Since *K* is the same for all days, this procedure compensates both the drift effects occurring during the measurements and the amplitude variations from day to day deriving from different conditions of the batteries and different temperatures of the antenna electronics. This is very important if we want to compare amplitudes measured during different days like before and after resin injections.

For the field data measured at the Carrara coast on the naturally fractured marble block, we followed the same data processing approach, i.e., we applied time calibration, dewow, bandpass filter to all records, and finally, we stacked the time-triggered records to obtain a single A-scan for any measurement position. In addition, three GPR calibration tests were measured using a metal shield placed on the opposite side of the block, in front of the antenna, to control the amplitude variations of the reflection. The test was repeated before and after each resin injection selecting three positions on the block that were not interfering with the fracture or the resin injections. The calibration factors were calculated with the same approach described for the laboratory measurements. 

Finally, the processing sequence that was applied to all the laboratory and field GPR data collected as continuous profiles by moving the antenna along the blocks (three profiles on the large block in laboratory and one profile on the naturally fractured block in Carrara) consisted of time calibration, dewow filter, bandpass filter, envelope extraction, and finally, amplitude calibration with the same procedure illustrated for the time-triggered measurements.

## 3. Results

### 3.1. Laboratory Measurements

**Figure 5:**
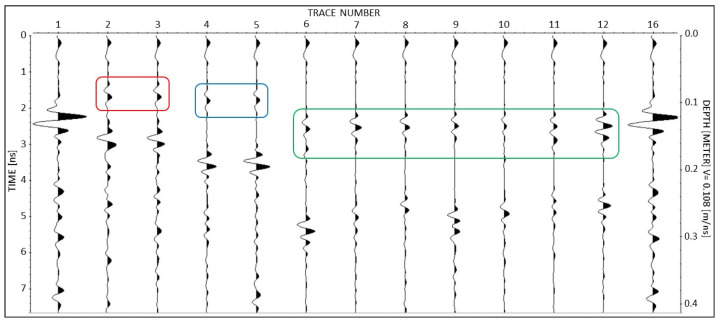
An example of a complete session of the laboratory time-triggered data after data processing and stacking. Traces 1 and 16 are the calibration records measured on the Botticino blocks. Traces 2 and 3 belong to the small blocks of Carrara. Traces 4 and 5 belong to the medium blocks. Traces 6–12 belong to the large blocks. The reflections of the artificial fractures are highlighted by three rectangles.

After stacking, all the time-triggered data of a measurement session, i.e., measurements related to the positions from 2 to 12 plus the calibration tests 1 and 16 (Figure 4a), were merged and compared on a single plot as shown in the example of Figure 5. The first and last traces relate to the Botticino blocks that were used for the calibration process. Trace numbers 2 and 3 showing a reflection at 1.2 ns (depth of about 6 cm) belong to the records of the small blocks of Carrara. Trace numbers 4 and 5 showing a reflection at 1.4 ns (depth of about 7 cm) belong to the records of the medium blocks. These pairs of records were measured in the same positions on the small and medium blocks using two orthogonal orientations of the antenna. Since the reflection came from the same reflecting point for each pair, the thin bed reflection was expected to show the same amplitude. On the contrary, for the records from 6 to 12 that belong to the large blocks and show a reflection at 2.2 ns (depth of about 11 cm), the reflecting position was changing, and this affected the amplitude of the reflection, as is clearly visible in Figure 5. 

The entire dataset consists of twenty-two sessions resulting from 11 days of measurements repeated every day with two different radar units. Therefore, twenty-two plots similar to Figure 5 have to be compared to analyze the radar sensitivity to resin injections.

The reflection amplitudes were measured by reading the peak-to-peak amplitude range of the reflected waves. This prevented any possible influence from a residual DC or a very low-frequency, unfiltered component of the signal. 

By using the amplitudes picked on traces 1 and 16, i.e., the amplitudes of the calibration tests, and following the procedure described above, all the data were calibrated to compensate the amplitude instabilities of the radar systems. By comparing the amplitude ranges measured with the calibration tests using radar unit 1 and radar unit 2, we concluded that radar unit 2 was less stable. Variations of ±14.03% were observed with radar unit 2, while radar unit 1 showed variations of ±9.77%. As a result, we decided to base our analysis only on the data produced by the best performing radar system and to ignore the data measured with the more unstable system.

The next step was to compare the calibrated amplitudes for all the time-triggered records measured after the beginning of resin injection (i.e., after the third day) with the corresponding amplitudes measured before the injections (e.g., with the measurements of the third day). The relative variation was calculated as:

(5) $${RV}_{d3\&j}=\left(\frac{{Amp}_{d3}-{Amp}_{j}}{{Amp}_{d3}}\right)\times 100$$

where ${RV}_{d3\&j}$ is the relative percentage variation of the amplitude between the *j*th day and day 3 (with *j* varying from 4 to 11), ${Amp}_{d3}$ is the amplitude on the third day, and ${Amp}_{j}$ is the amplitude on the *j*th day. Using this definition, only positive variations were expected because resin injections would reduce the reflected amplitude. Negative variations, if observed, were attributed to noise or residual drift effects.

Table 2 shows the percentage variations measured for the small and medium blocks averaging the eight measurement days (from day 4 to day 11) because the fracture was fully injected between days 3 and 4, and no resin variations occurred after that. Since the rotation of the antenna was not expected to affect the amplitudes reflected by the planar fractures, each average included both measurements performed on each block, i.e., measurements 2 and 3 for the small blocks and measurements 4 and 5 for medium blocks (Figure 4a). Thus, the average was calculated using the following equation for each block:

(6) $$\bar{RV}=\frac{1}{8}\sum_{j=4}^{11}\left(\frac{{RV}_{d3\&j}^{\to }+{RV}_{d3\&j}^{↑}}{2}\right)$$

where $\bar{RV}$ is the average percentage variation measured on the small or medium blocks, and ${RV}_{d3\&j}^{\to }$ and ${RV}_{d3\&j}^{↑}$ are the relative percentage variations of the amplitude between the *j*th day and the third day, measured with the horizontal and vertical orientation of the antenna, respectively. Table 2 also reports the standard deviations to demonstrate that the residual drift effects and data noise did not remarkably affect the stability and significance of the amplitude variations observed on different days.

**Figure 6:**
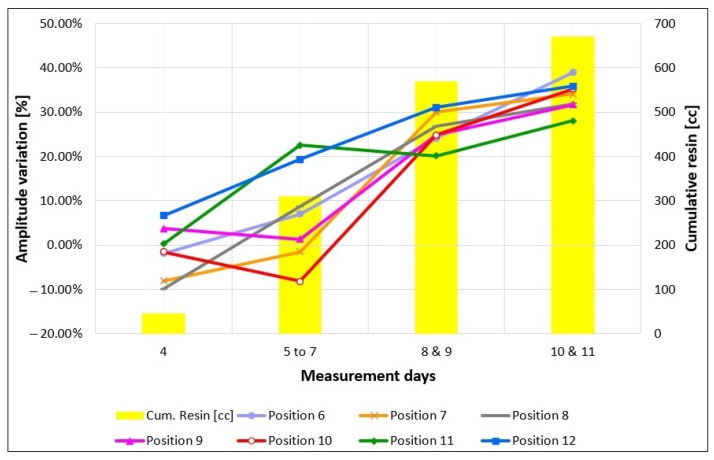
Amplitude percentage variations of the artificial fracture reflections measured on the large block at the selected positions after each injection phase. Positive numbers indicate amplitude decrease. Yellow bars indicate the cumulative amount of the resin injected after each phase.

Figure 6 shows the percentage variations measured after each injection phase on the seven different positions tested on the large blocks (positions 6–12 shown in Figure 4a). Except for the data points measured on the fourth day after the first injection of only 45 cc of resin, other data points are the average values of the measurements repeated on successive days, i.e., three times after the second injection, and two times after the third and the fourth injections. The yellow bars indicate the cumulative resin amount resulting after each injection phase.

**Figure 7:**
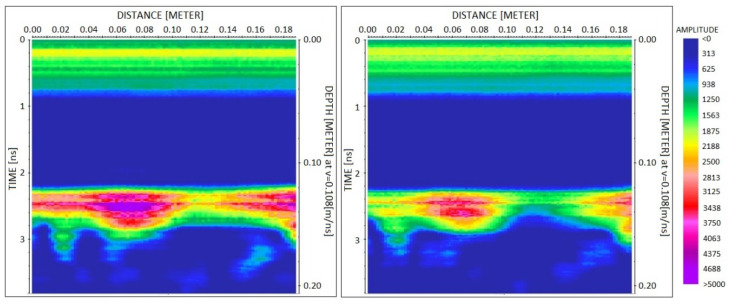
Comparison of profile 14 measured on the large specimen before and after injections on the third day (**left**) and on the last day (**right**), respectively. Data are plotted after time and amplitude calibration, dewow filter, and envelope extraction.

**Figure 8:**
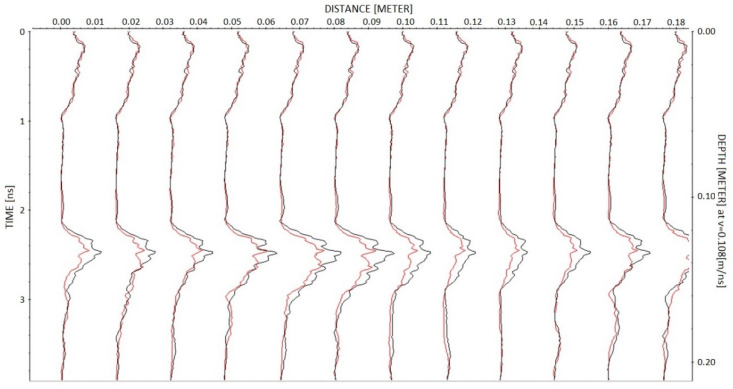
Data of Figure 7 overlapped after decimation by a factor of eight along the horizontal axis. In black, the data before injections, and in red, the data after injections.

To illustrate the results obtained with the profiles 13, 14 and 15 measured by moving the antenna on the large specimen, Figure 7 shows the comparison of profile 14 measured on the third day, i.e., before injections (left image), with profile 14 measured on the last day, i.e., after full injection of the fracture (right image). Data are presented after applying the dewow filter and after time and amplitude calibration, by plotting the radar trace envelope with the same color scale to facilitate the amplitude comparison of the reflection from the fracture arriving at about 2.2 ns. For a more detailed comparison of the reflected amplitudes, a selection of the same data (one trace every eight traces), is compared in Figure 8 by overlapping the trace envelope before and after injections. Very similar images were also observed by analyzing profiles 13 and 15 measured before and after injections. 

### 3.2. Field Measurements

**Figure 9:**
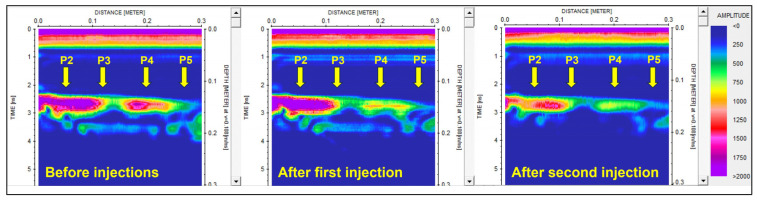
The radar profile measured on the fractured block on the Marina di Carrara before and after the two injection phases. Data are plotted after time and amplitude calibration, dewow filter, and envelope extraction. The arrows indicate the approximate positions of the point measurements reported in Figure 10.

**Figure 10:**
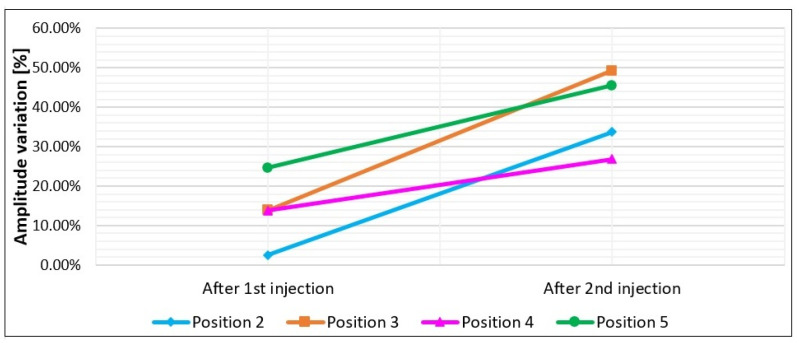
Amplitude percentage reductions of the natural fracture reflection measured at the selected positions after the first and the second injection phases. Position 6 is not reported because the fracture reflection was interfering with a strong diffraction generated by the metal element embedded in the block.

Field data measured on the selected block in Marina di Carrara were processed using the same processing steps applied to the laboratory data. The vertical wheel-driven profile is shown in Figure 9 to compare the envelope amplitudes of the reflection before injections, after the first injection phase, and finally, after the concluding injection phase. The arrival time of the fracture reflection was 2.2 ns (about 12 cm) at the beginning of the profile (i.e., at the top of the block) and gently increased as the antenna moved down along the block face. At 30 cm from the initial position, the apparent depth of the fracture was about 14 cm. The profile was cut at 30 cm because the last part was disturbed by the diffraction produced by the metal target embedded in the block (Figure 2e). For the same reason, we analyzed the point data measured in positions 2 to 5 (Figure 4b), while we ignored position 6 which was too close to the metal element. The percentage amplitude reductions measured at these positions after the first and the second injections are illustrated in Figure 10. To compare the profile amplitude variations with the point measurements, Figure 9 shows the approximate positions of the selected points. 

## 4. Discussion

### 4.1. Laboratory Measurements

**Figure 11:**
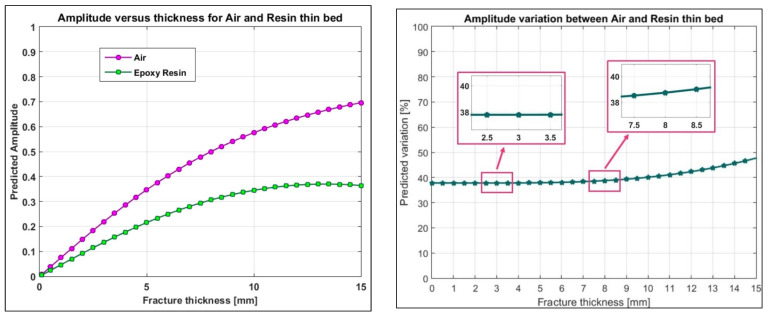
Comparison of the predicted amplitudes for the air-filled fractures (purple circles) versus the resin-filled fractures (green squares) for a 3 GHz antenna. Marble relative permittivity: 7.7. Resin relative permittivity: 3.5. The right plot shows the percentage amplitude reduction expected when the resin substitutes the air within the fracture.

Numerical simulations and analytical models can predict the difference between the air-filled and resin-filled fractures [18,21]. Figure 11 indicates the predicted theoretical difference between the air-filled and the resin-filled fractures obtained by tuning the analytical models to the actual marble velocity measured on the Carrara blocks (10.8 cm/ns). We assumed the relative permittivity of 3.5 for the resin. This is the minimum value of the expected range for resin (3.5 to 4.7) and thus, the results will illustrate the minimum theoretical differences that we can predict before and after injections. Based on Figure 11, if GPR detects an air-filled fracture, a minimum of 38% amplitude reduction is expected after the fracture is filled with the resin. In our laboratory tests, the predicted variation is about 38% for the 3 mm-thick fractures (the average fracture thickness for the small and medium blocks), and 39% for the 8 mm-thick fracture (the average fracture thickness for the large block). This percentage variation will increase to about 48% for thicker fractures (15 mm).

By observing the percentage amplitude variations reported in Table 2, we can conclude that the effect of resin injections within the fracture has been successfully detected for both the small and the medium specimens. For the medium block, the average variation is very close to the expected theoretical value (around 38%), while for the small block, the variation is underestimated but still clearly assessed because a reduction of 25.59% in reflection amplitude is so remarkable that it cannot be attributed to noise or to antenna power instabilities that were not properly compensated by the calibration procedure.

By observing the graph in Figure 6, we understand that the first initial injection was so minimal (only 45 cc) that was not able to fill the fracture in any position, as proved by the GPR amplitude variations that are negative or close to 0% with a maximum positive value of 6.65% in position 12. Note that negative variations are obviously not attributable to the presence of the resin because resin injections cannot produce an amplification of the reflected wave. Thus, negative variations are the result of noise and residual drift effects. On the contrary, positive variations could be attributed to resin injections, but the values are too far from the theoretical expectations (about 38%) associated with a filled fracture. Moreover, positive variations never exceed 7% which is almost the standard deviation observed on the medium block test. Therefore, the positive variations are very likely attributable to noise and residual drift effects as well. After the second injection, resulting in a cumulative resin volume of 310 cc, the situation does not change for most positions except for positions 11 and 12 in which the amplitude variations rise to values close to, or a little larger than 20%, suggesting that the resin was mostly flowing towards these two positions. This is consistent with the expectations based on the inclination given to the specimen. A 20% variation cannot be confused with amplitude variations produced by noise or residual drift effects. Thus, it must be caused by resin injections; however, there might be the probability that the fracture was still not totally filled. After another injection, resulting in a cumulative resin volume of 570 cc, all amplitude variations approached values between 20% and 31%. This proves that resin has reached all the tested positions; however, in some points where the variations are still less than 25%, we might think that the filling is not complete. It is interesting to note that the amplitude variation at position 12 jumps up from about 19% to 31%, the highest value measured at this stage. This seems to confirm that the inclination of the specimen produces a preferential flow directed towards that position, which seems to be the first zone to be totally filled. After the last injection, resulting in a cumulative resin volume of 670 cc, all amplitude variations show further small increases, with all values in the range 28–39%. According to GPR, we conclude that the injections were successful at all positions. This is consistent with the fact that at this stage, no more resin could be injected inside the fracture, and we are mostly sure that the fracture was completely filled everywhere. It is interesting to note that the amplitude variation at position 6 moves from 24% to 39% at the final stage. This is the highest increase observed between stage three and stage four. Considering that the injection point was close to position 6, and that the specimen was gently inclined to favor the resin flow towards the opposite side of the block, it is quite reasonable that position 6 was not totally filled after stage three and was completely filled only after the final injection.

With the perspective of a real application where the success of resin injections must be checked rapidly and extensively, GPR profiles are preferable to single-point measurements. This is the reason why we also performed some GPR profiles on the larger specimen, where the antenna could run for about 20 cm. The comparison of profiles measured before and after injections proves the effectiveness of the approach. By observing Figure 7 or Figure 8, it is easy to assess the success of injections along the entire distance covered by the profile. Apparently, Figure 8 shows amplitude variations in the order of 30% before and after injections. This means that the amplitude reduction is clear enough and cannot be misinterpreted as a random error produced by noise or residual drift effects. However, later in this section, we will analyze these data more quantitatively to illustrate how continuous profiles can be automatically interpreted for injection assessment. 

### 4.2. Field Measurements

The GPR data from the marble block with a natural fracture on Carrara coast were analyzed to validate the injection assessment procedure on a real case. The natural fracture is expected to be irregular in terms of geometry and thickness. These irregularities are not an issue provided that the measurements before and after injections are accurately repeated in the same positions or exactly along the same trajectories, so that the comparison of reflection amplitudes before and after injections is meaningful [21]. 

The fracture reflection recorded along the vertical profile (see Figure 4b for profile location) is illustrated in Figure 9 and shows a progressive amplitude reduction. After the first injection, it seems that the first 12 cm of the profile are not affected by resin, while from 12 cm down to 30 cm, we observe a clear amplitude reduction suggesting that resin has started to fill the middle and lower parts of the fracture. We remind here that the profile runs vertically from top to bottom along the block side that faces the fracture, and that the fracture was filled by injecting the resin from the top aperture. Thus, it is obvious to expect resin accumulation in the lower part of the block that corresponds to the last part of the radar profile. After the second injection that apparently was able to completely fill the natural fracture, the profile shows a further reduction of the reflected amplitudes in the middle and lower parts of the fracture, but also a remarkable amplitude decrease is observed in the upper part. These results are fully consistent with the expectations. To have a more quantitative evaluation of the resin effects, we can analyze the amplitude percentage reductions reported in Figure 10. After the first injection, position 5 (the one in the lowest location excluding position 6 for the mentioned interference of the embedded metal element) shows a remarkable amplitude reduction of about 25%, which certainly indicates the arrival of some resin at this position. After the second injection, this position records another remarkable decrease in amplitude, arriving at a final percentage reduction of about 45%, which should correspond to the complete filling of the fracture with the resin. For positions 3 and 4, we observe a discrete decrease in the amplitude (about 14%) after the first injection, which probably should be interpreted as an initial, but limited, filling of the fracture occurring in the middle part of the block. A reduction of 14% is too high to be attributed to noise or residual drift effects, but it is also too low to indicate the complete filling of the fracture. After the second injection, positions 3 and 4 both report a further remarkable reduction, arriving at about 49% and 27%, respectively, values that are consistent with the complete filling of the fracture. Finally, at position 2, we do not observe any significative variations after the first injection (the reduction of about 2% is surely due to noise or residual drift effects), while we observe a very sudden change after the second injection with a final amplitude reduction of about 34%. On the whole, the behaviors observed at the selected positions are consistent with what was qualitatively observed along the GPR profile (Figure 9), and demonstrate that the first injection, although able to produce preliminary amplitude effects in the middle and lower parts of the block, was not fully sealing the fracture. On the contrary, the fracture was fully sealed with the second injection, and at all the positions, the expected amplitude reduction was recorded with large values, up to 49% at position 3, and never less than 27% at the other positions. The large range of the amplitude reduction recorded along the block is very likely due to the thickness variations of the fracture. According to visual inspections of the external faces of the block, the fracture aperture varies from 1 mm to 10 mm (Figure 2c,d), but we have no idea about the real aperture inside the block where the GPR signal was actually reflected. According to analytical predictions (Figure 11), the largest variations (49%) could indicate a fracture aperture of about 15 mm or slightly more, but we must be honest in saying that noise and residual drift effects can introduce important errors (up to about 10%, according to what we observed with the laboratory tests). Thus, while the accuracy offered by this procedure does not preclude the possibility to assess the success of the resin injections, the measurement of the fracture thickness based on the observed injection effects is not accurate enough, and only a very rough indication about thickness can be derived as an optional add-on of the procedure.

### 4.3. Automatic Analysis

**Figure 12:**
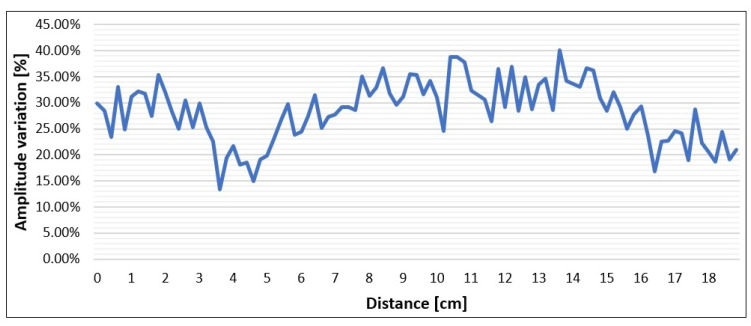
Percentage reduction in the reflected amplitude automatically picked along profile 14 measured on the large specimen before and after resin injections (third day and last day respectively).

In the perspective of an industrial application of the GPR method for the assessment of the success of resin injections, the procedure must be reliable but also cost-effective, i.e., rapid and easy. The above laboratory and field tests have proved that the methodology is reliable in discriminating where the fracture has been sealed from where the resin has not arrived or has not completely filled the aperture. Based on our tests, a reduction percentage of 20% may be the threshold that discriminates between successful and unsuccessful injection, while 10% may be the threshold that discriminates between partial filling and no resin at all in the fracture. In order to use these quantitative indicators, not only at some check points where we decide to deploy the antenna and to collect a time-triggered survey, but on the whole surface of the block where we can run profiles that investigate the expected fracture, we need to develop an automatic procedure for the extensive analysis of the amplitude variations. The procedure must consist of an automatic amplitude picking of the highest amplitudes measured on the envelope of the fracture reflections, before and after injections, and in the calculation of the relative percentage variation. In principle, the procedure is very simple and the amplitude analysis algorithm is not a problem at all. The main issue in applying the suggested procedure is actually on the data acquisition side because any profile before and after injections must be rigorously repeated by moving the antenna along the same trajectory. This is particularly important when the fracture thickness is highly variable. To make the algorithm simpler, a good suggestion would be to collect the data with the same spatial sampling interval, so that no decimation or interpolation are needed to compare the profiles. To test a prototype of this automatic algorithm, we used the short profiles measured in laboratory on the large specimen. These profiles were collected with the required accuracy both regarding precise positioning and calibration tests, and so the direct quantitative comparison of the profiles before and after injections was possible. Figure 12 shows the result of the amplitude variation indicator along the full trajectory of profile 14, the same profile presented in Figure 7 and Figure 8. The spatial sampling of the indicator is the same as the original radar profile, i.e., 2 mm. Most values are above the threshold of 20% and assess the success of the injections along 90% of the profile, as was qualitatively supposed by looking at Figure 7. However, a few values around the distance of 4 cm from the beginning of the profile and at the end of the profile fall in the range of 14–20% which might suggest that very minor air gaps are still remaining at these positions. These points were not sampled by the measurements in the fixed positions, for which we observed high amplitude reductions everywhere (Figure 6). It is interesting to note that the distance intervals where Figure 12 seems to indicate partial filling problems correspond to locations where the reflection received from the fracture is quite strong (Figure 7). A possible interpretation is that the high amplitudes of the reflection at these locations compared to the lower amplitudes of other sections of the profile are due to larger apertures of the fracture which, for this specimen, is expected to vary between 1 mm and 15 mm. Considering that the resin flow within the fracture was produced by gravity without applying any pressure to the fluid, and that the specimen was only gently lifted on the injection side to favor the resin flow in the desired direction, given the fact that the two overlapped blocks were only deployed one over the other without any mechanical connection, it is quite possible that the resin fluid did not completely fill the gap in some points where the fracture was a little thicker, especially when this was due to irregularities of the lower surface of the upper block. On the whole, the entire procedure, consisting of careful acquisitions of radar profiles and specific calibration tests followed by proper data processing and an automatic algorithm for amplitude picking and analysis, seems sufficiently reliable and cost-effective for the purpose of quality control of the resin injections.

## 5. Conclusions

In this study, GPR was tested as a promising method for non-destructive quality control of resin injections used to repair marble blocks prior to their use in the ornamental or construction industries. The proposed procedure, consisting of time-lapse measurements properly performed before and after injections, was validated through laboratory tests on three artificially fractured specimens and through a field test on a naturally fractured marble block. For the injections, we used epoxy resin, which is a preferred filling material in the stone market. The principle of the quality control of injections is the comparison of the reflected amplitudes received from the fracture before and after injections. The interpretation of the results was guided by the expectations based on analytical predictions valid under the assumption that the fracture behaves like a thin bed. According to the thin bed theory, a resin injected fracture investigated with a 3 GHz antenna behaves like a thin bed up to a thickness of about 12–14 mm, the precise value depending on the actual permittivity of the resin. Our laboratory and field tests were performed on fractures with thicknesses varying from 1 mm to 15 mm, and the result was that the method was always able to assess the injection success or failure. 

Based on our experimental results and the theoretical predictions, we could calibrate two amplitude variation thresholds (10% and 20%) that discriminate between the three following situations:

- When the amplitude variation is lower than 10%, no resin is filling the fracture and it shows an unsuccessful injection.
- When the amplitude variation is in the range 10% to 20%, it shows the partial filling of the fracture, i.e., resin could only reduce the fracture thickness without filling it completely.
- The amplitude variations higher than 20% show a successful injection, meaning that the resin has fully sealed the fracture.

An optional add-on of the procedure is the evaluation of the fracture thickness at any measurement point, though with the tested equipment, for which power instabilities cannot be ignored, we have to admit that the accuracy of this value is still very rough. As a matter of fact, antenna power instabilities and drifts are still the bottleneck of the entire procedure and the point on which to work in the future to improve the accuracy and make the test easier. Currently, power instabilities do not prevent the application of the method, but they require a proper calibration procedure during any test. The method was successfully tested both with point measurements and with profile measurements, and an automatic algorithm was proposed and validated for quantitative evaluations along the profiles.

As expected, the difference between the air and the resin within the fracture was clearly detectable by a high-frequency GPR antenna, and we can conclude that the GPR method is very promising in the marble industry for assessing the fracture condition before and after treatment, thus reducing the waste of natural stone reserves by improving the effectiveness of repair interventions.

## Figures and Tables

**Table 1:** Details for fracture conditions during the eleven days of laboratory measurements. Injections were always performed at the end of the day after the GPR measurements of that day.

	Small Blocks		Medium Blocks		Large Blocks	
Day	Fracture Filler	Injections [cc]	Fracture Filler	Injections [cc]	Fracture Filler	Injections [cc]
1st	Air		Air		Air	
2nd	Air		Air		Air	
3rd	Air		Air		Air	
		101		163		45
4th	Resin		Resin		Air	
						265
5th	Resin		Resin		Air+Resin (310 cc)	
6th	Resin		Resin		Air+Resin (310 cc)	
7th	Resin		Resin		Air+Resin (310 cc)	
						260
8th	Resin		Resin		Air+Resin (570 cc)	
9th	Resin		Resin		Air+Resin (570 cc)	
						100
10th	Resin		Resin		Resin	
11th	Resin		Resin		Resin	

**Table 2:** Average percentage reductions and standard deviations measured on the fractures of the small and medium blocks after resin injections.

	Small Blocks	Medium Blocks
**Average variation**	25.59%	33.64%
**Standard deviation**	3.62%	7.43%

## Data Availability

Datasets used in this research will be sent to interested researchers upon request.

## References

[1] Ashmole I., Motloung M. (2008). Dimension Stone: The Latest Trends in Exploration and Production Technology.

[2] Egesi N., Tse C.A. (2011). Dimension stone: Exploration, evaluation and exploitation in southwest parts of Oban Massif Southeastern Nigeria. J. Geol. Min. Res..

[3] Jug J., Grabar K., Strelec S., Dodigović F. (2020). Investigation of dimension stone on the Island Brač—Geophysical approach to rock mass quality assessment. Geosciences.

[4] Sousa L., Barabasch J., Stein K.-J., Siegesmund S. (2017). Characterization and quality assessment of granitic building stone deposits: A case study of two different Portuguese granites. Eng. Geol..

[5] Torkan M., Janiszewski M., Uotinen L., Baghbanan A., Rinne M. (2022). Photogrammetric method to determine physical aperture and roughness of a rock fracture. Sensors.

[6] Hojat A. A review of the last decade of Ground Penetrating Radar contribution to the marble quarrying industry (Invited Talk). Proceedings of the 5th Asia Pacific Meeting on Near Surface Geoscience & Engineering.

[7] Zanzi L., Hojat A., Ranjbar H., Karimi-Nasab S., Azadi A., Arosio D. (2019). GPR measurements to detect major discontinuities at Cheshmeh-Shirdoosh limestone quarry, Iran. Bull. Eng. Geol. Environ..

[8] Smith M.R. (1999). Stone: Building Stone, Rock Fill and Armourstone in Construction.

[9] Yarahmadi R., Bagherpour R., Taherian S.-G., Sousa L.M.O. (2019). A new quality factor for the building stone industry: A case study of stone blocks, slabs, and tiles. Bull. Eng. Geol. Environ..

[10] Hojat A., Izadi-Yazdanabadi M., Karimi-Nasab S., Arosio D., Zanzi L. GPR method as an efficient NDT tool to characterize carbonate rocks during different production stages. Proceedings of the EAGE-GSM 2nd Asia Pacific Meeting on Near Surface Geoscience & Engineering.

[11] Grandjean G., Gourry J.-C. (1996). GPR data processing for 3D fracture mapping in a marble quarry (Thassos, Greece). J. Appl. Geophys..

[12] Lualdi M., Zanzi L. 2D and 3D experiments to explore the potential benefit of GPR investigations in planning the mining activity of a limestone quarry. Proceedings of the 10th International Conference on Ground Penetrating Radar GPR2004.

[13] Arosio D., Hojat A., Munda S., Zanzi L. Non-destructive root mapping: Exploring the potential of GPR. Proceedings of the 3rd Asia Pacific Meeting on Near Surface Geoscience & Engineering.

[14] Hojat A., Ranjbar H., Karimi-Nasab S., Zanzi L. (2023). Laboratory tests and field surveys to explore the optimum frequency for GPR surveys in detecting qanats. Pure Appl. Geophys..

[15] Arosio D., Hojat A., Munda S., Zanzi L. High-frequency GPR investigations in San Vigilio Cathedral, Trento. Proceedings of the 24th European Meeting of Environmental and Engineering Geophysics.

[16] Binda L., Lualdi M., Saisi A., Zanzi L. (2011). Radar investigation as a complementary tool for the diagnosis of historic masonry buildings. Int. J. Mater. Struct. Integr..

[17] Barraca N., Almeida M., Varum H., Almeida F., Matias M.S. (2016). A Case study of the use of GPR for rehabilitation of a classified art deco building: The InovaDomus House. J. Appl. Geophys..

[18] Arosio D. (2016). Rock fracture characterization with GPR by means of deterministic deconvolution. J. Appl. Geophys..

[19] Arosio D., Zanzi L., Longoni L., Papini M. (2013). GPR investigations of rock fractures: Considerations on thin beds. Symposium on the Application of Geophysics to Engineering and Environmental Problems 2013.

[20] Arosio D., Zanzi L., Longoni L., Papini M. Fracture thickness from GPR measurements. Proceedings of the 8th International Workshop on Advanced Ground Penetrating Radar (IWAGPR).

[21] Izadi-Yazdanabadi M., Hojat A., Zanzi L., Karimi-Nasab S., Arosio D. (2022). Analytical models and laboratory measurements to explore the potential of GPR for quality control of marble block repair through resin injections. Appl. Sci..

[22] Arosio D., Munda S., Zanzi L. Quality control of stone blocks during quarrying activities. Proceedings of the 14th International Conference on Ground Penetrating Radar.

[23] Grégoire C., Hollender F. (2004). Discontinuity characterization by the inversion of the spectral content of Ground Penetrating Radar (GPR) reflections—Application of the Jonscher model. Geophysics.

[24] Markovaara-Koivisto M., Hokkanen T., Huuskonen-Snicker E. (2014). The effect of fracture aperture and filling material on GPR signal. Bull. Eng. Geol. Environ..

[25] Sambuelli L., Calzoni C. (2010). Estimation of thin fracture aperture in a marble block by GPR sounding. Boll. Geof. Teor. Appl..

[26] Shakas A., Linde N. (2015). Effective modeling of ground penetrating radar in fractured media using analytic solutions for propagation, thin-bed interaction and dipolar scattering. J. Appl. Geophys..

[27] Orlando L., Slob E. (2009). Using multicomponent GPR to monitor cracks in a historical building. J. Appl. Geophys..

[28] Ortega-Ramírez J., Bano M., Larrea-López L.L., Robles-Camacho J., Ávila-Luna P., Villa-Alvarado L.A. (2019). GPR measurements to identify cracks and textural arrangements in the altar wall of the 16th-century Santa Maria Huiramangaro Church, Michoacán, Mexico. Surf. Geophys..

[29] Molron J., Linde N., Baron L., Selroos J.-O., Darcel C., Davy P. (2020). Which fractures are imaged with ground penetrating radar? results from an experiment in the Äspö hardrock laboratory, Sweden. Eng. Geol..

[30] Seren A., Demirkol Acikgoz A. (2012). Imaging fractures in a massive limestone with ground penetrating radar, Haymana, Turkey. Sci. Res. Essays.

[31] Grasmueck M. (1996). 3-D ground-penetrating radar applied to fracture imaging in gneiss. Geophysics.

[32] Isakova E.P., Daniliev S.M., Mingaleva T.A. (2021). GPR for mapping fractures for the extraction of facing granite from a quarry: A case study from Republic of Karelia. E3S Web Conf..

[33] Johnston B.J., Ruffell A., Warke P., McKinley J. (2019). 3DGPR for the non-destructive monitoring of subsurface weathering of sandstone masonry. Heritage.

[34] Elkarmoty M., Tinti F., Kasmaeeyazdi S., Bonduà S., Bruno R. (2018). 3D modeling of discontinuities using GPR in a commercial size ornamental limestone block. Constr. Build. Mater..

[35] Annan A.P. (2001). Ground Penetrating Radar Workshop Notes.

[36] Deparis J., Garambois S. (2009). On the use of dispersive APVO GPR curves for thin-bed properties estimation: Theory and application to fracture characterization. Geophysics.

[37] Jol H.M. (2008). Ground Penetrating Radar Theory and Applications.

[38] Bradford J.H., Deeds J.C. (2006). Ground-Penetrating Radar theory and application of thin-bed offset-dependent reflectivity. Geophysics.

[39] Arosio D., Deparis J., Zanzi L., Garambois S. Fracture characterization with GPR: A comparative study. Proceedings of the 16th International Conference on Ground Penetrating Radar.

[40] Ashurst J., Dimes F.G. (1998). Conservation of Building and Decorative Stone.

[41] Arndt B., DeMarco M., Andrew R. (2008). Polyurethane Resin (PUR) Injection for Rock Mass Stabilization.

[42] López-Buendía A.M., Guillem C., Cuevas J.M., Mateos F., Montoto M. (2013). Natural stone reinforcement of discontinuities with resin for industrial processing. Eng. Geol..

[43] Baraka-Lokmane S. (2002). A new resin impregnation technique for characterising fracture geometry in sandstone cores. Int. J. Rock Mech. Min. Sci..

[44] De Rosario I., Elhaddad F., Pan A., Benavides R., Rivas T., Mosquera M.J. (2015). Effectiveness of a novel consolidant on granite: Laboratory and in situ results. Constr. Build. Mater..

[45] Pinto A.P.F., Rodrigues J.D. (2008). Stone consolidation: The role of treatment procedures. J. Cult. Herit..

[46] Selwitz C. (1992). Epoxy Resins in Stone Conservation.

[47] Demirdag S. (2009). The effect of using different polymer and cement based materials in pore filling applications on technical parameters of travertine stone. Constr. Build. Mater..

[48] Clifton J.R. (1980). Stone Consolidating Materials: A Status Report.

[49] Selwitz C. The use of epoxy resins for stone consolidation. Proceedings of the Material Issues in Art & Archeology II.

[50] Tesser E., Lazzarini L., Bracci S. (2018). Investigation on the chemical structure and ageing transformations of the cycloaliphatic epoxy resin EP2101 used as stone consolidant. J. Cult. Herit..

[51] Hosseini M., Karapanagiotis I. (2018). Advanced Materials for the Conservation of Stone.

[52] Terreni & COA s.r.l. http://terreniecoa.it/en/quarries/.

[53] IDS GeoRadar. https://idsgeoradar.com/products/ground-penetrating-radar.

[54] Sandmeier D.K.J. REFLEXW—GPR and Seismic Processing Software. https://www.sandmeier-geo.de/reflexw.html.

